# Combined ^31^P and ^1^H MRS show that impaired muscle O_2_ supply limits ATP synthesis during exercise in systemic sclerosis

**DOI:** 10.1042/CS20261467

**Published:** 2026-08-27

**Authors:** Graham J. Kemp

**Affiliations:** Institute of Life Course and Medical Sciences, Department of Musculoskeletal and Ageing Science, University of Liverpool, William Henry Duncan Building, 6 West Derby Street, Liverpool, L7 8TX, U.K.

**Keywords:** 1H MRS, 31P MRS, deoxymyoglobin, mitochondria, Muscle energetics

## Abstract

Muscle weakness and fatigue feature in many diseases, but the underlying pathophysiology can be difficult to study *in vivo*. In patients with the chronic autoimmune disease systemic sclerosis (SSc), considerable disability results from small-vessel vasculopathy and progressive fibrosis. Alongside cardiopulmonary limitations on exercise capacity, there is increasing evidence of peripheral dysfunction. In a study reported in this issue, Layec and colleagues investigate this in exercising skeletal muscle *in vivo* using a combination of phosphorus magnetic resonance spectroscopy (^31^P MRS) and proton magnetic resonance spectroscopy (^1^H MRS). The ^31^P MRS abnormalities (a bigger change in phosphocreatine (PCr) concentration during exercise and slower post-exercise PCr recovery) reflect impaired mitochondrial ATP synthesis, and the larger change in deoxymyoglobin concentration measured by ^1^H MRS (implying lower myocyte PO_2_) implicates a defect in O_2_ supply rather than in mitochondrial O_2_ use. This major contributor to impaired exercise tolerance would be difficult to establish using classical methods of biopsy and exercise physiology, which shows the power of non-invasive measurement modalities to probe the pathophysiology of complex conditions.

An invited Commentary on ‘Compromised muscle energetics and intracellular O_2_ availability during dynamic contraction in the plantar flexor muscles of patients with systemic sclerosis’.

The present study combines the two non-invasive methods of ^31^P MRS and ^1^H MRS to probe the interface between muscle metabolism and vascular physiology *in vivo* (see Figure 1). The earliest *in vivo* application of ^31^P MRS was in skeletal muscle, where what it can detect directly (broadly, mobile tissue phosphates at >0.1 mM) includes some key participants in muscle energetics (Pi, PCr, and ATP) and allows calculation of several others (cytosolic pH, free [ADP], and ΔG_ATP_) [1]. Despite some technical challenges (mainly of sensitivity, localisation and quantitation), the ability to acquire time-series data during exercise and post-exercise recovery offers a unique window on metabolism related to ATP turnover [2,3], especially in clinical research on subjects with limited tolerance for invasive techniques [1]. ^31^P MRS data from working muscle *in vivo*, appropriately interpreted in terms of the underlying physiology and metabolism, can be used to quantify ATP use for force generation (and thus contractile cost), ATP production by oxidative phosphorylation and by glycogenolysis, and processes influencing cytosolic pH [2,3].

**Figure 1:**
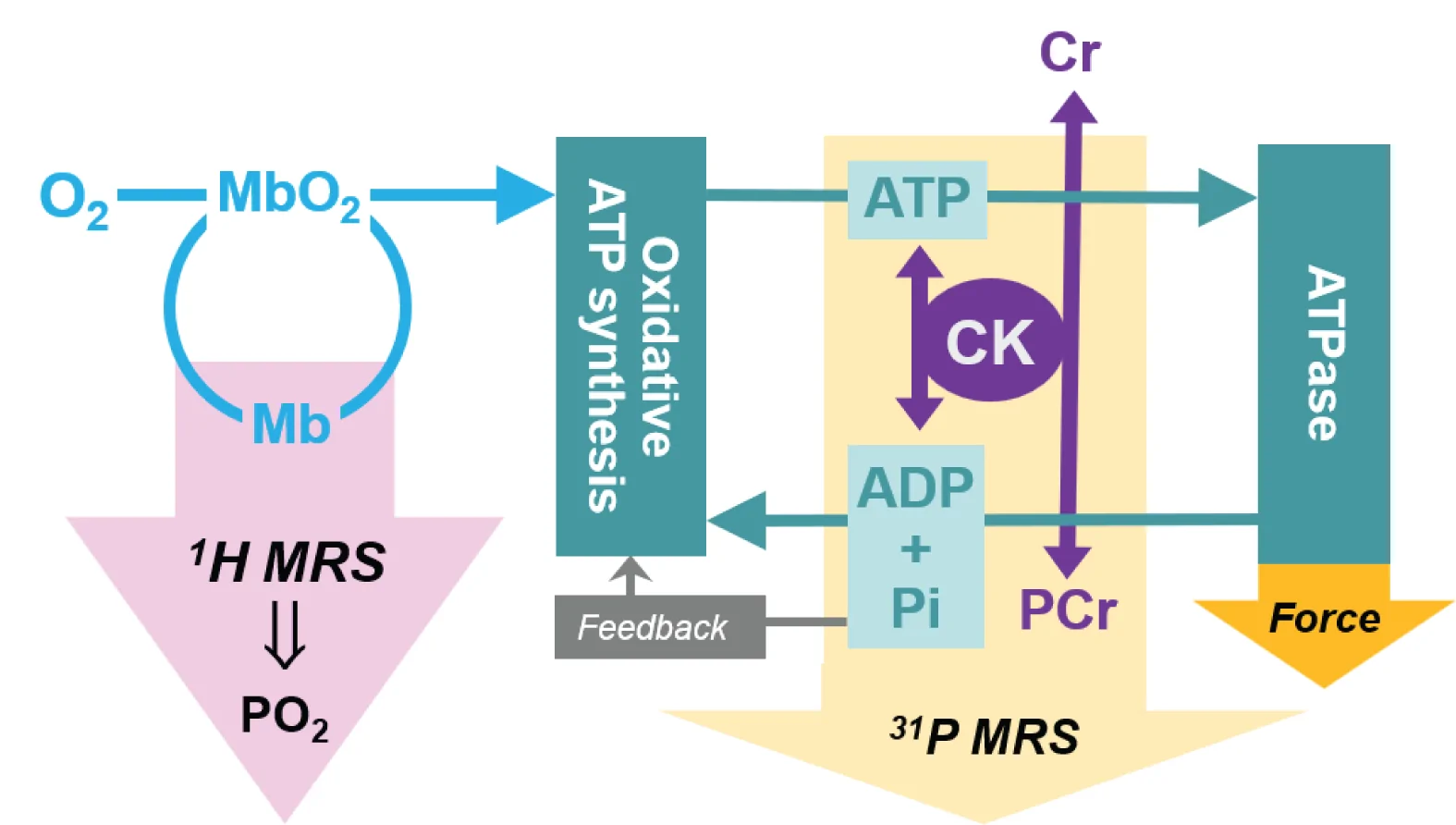
ATP and oxygen turnover in exercising muscle as seen by ^31^P and ^1^H MRS Muscle energy metabolism is simplified to ATP supply by oxidative phosphorylation and ATP use by the myosin ATPase to generate measurable mechanical force. ADP and inorganic phosphate (Pi) are substrates for the former and products of the latter. The creatine kinase (CK) reaction equilibrates phosphocreatine (PCr), creatine (Cr), ATP, ADP, and H^+^, such that any temporary mismatch between ATP supply and demand is buffered by PCr breakdown. In this relatively low-power exercise, glycolytic ATP synthesis can be neglected: for a fuller picture see [3]. ATP turnover is largely demand-driven, and CK-related metabolites (notably [Pi] and [ADP], and the free energy of ATP hydrolysis (ΔG_ATP_) to which they contribute) are important feedback regulators matching ATP supply to ATP use. To supply oxidative ATP synthesis, O_2_ diffuses in from vascular HbO_2_, and travels within the cell to the mitochondrion largely as MbO_2_. ^31^P MRS (pale orange arrow) can quantify the key metabolites shown. ^1^H MRS (pink arrow) can detect deoxymyoglobin, from which myocyte PO_2_ can be calculated. ^1^H MRS can also measure several other relevant muscle metabolites [15].

The study makes use of three ^31^P MRS-based measurements: the initial-exercise rate of PCr fall, to measure contractile cost (which is normal in SSc); the subsequent PCr fall, reflecting ATP supply-demand mismatch (higher in SSc); and the initial post-exercise PCr resynthesis rate, a measure of end-exercise oxidative ATP synthesis rate (lower in SSc) [4]. This impaired oxidative ATP synthesis has two potential causes [5]: defective O_2_ use caused by mitochondrial abnormalities (e.g. low mitochondrial density or impaired/deficient components of oxidative metabolism), and defective O_2_ supply (due to macro- or micro-vascular dysfunction or impaired O_2_ diffusion). To distinguish these, ^1^H MRS is used to measure deoxymyoglobin, and thus estimate myocyte PO_2_ [4]. Figure 1 shows how the two techniques combine to give a picture of the whole O_2_ transport and usage chain.

In general the kinetics of PO_2_ reflect the dynamic balance between O_2_ supply and O_2_ use [4], but the use here is straightforward: if mitochondrial ATP synthesis in SSc is limited by O_2_ supply, then cellular PO_2_ will be lower (as indeed it is). Figure 2 attempts to put this clear result and implication into a wider physiological context, in order to suggest some avenues for future work.

**Figure 2:**
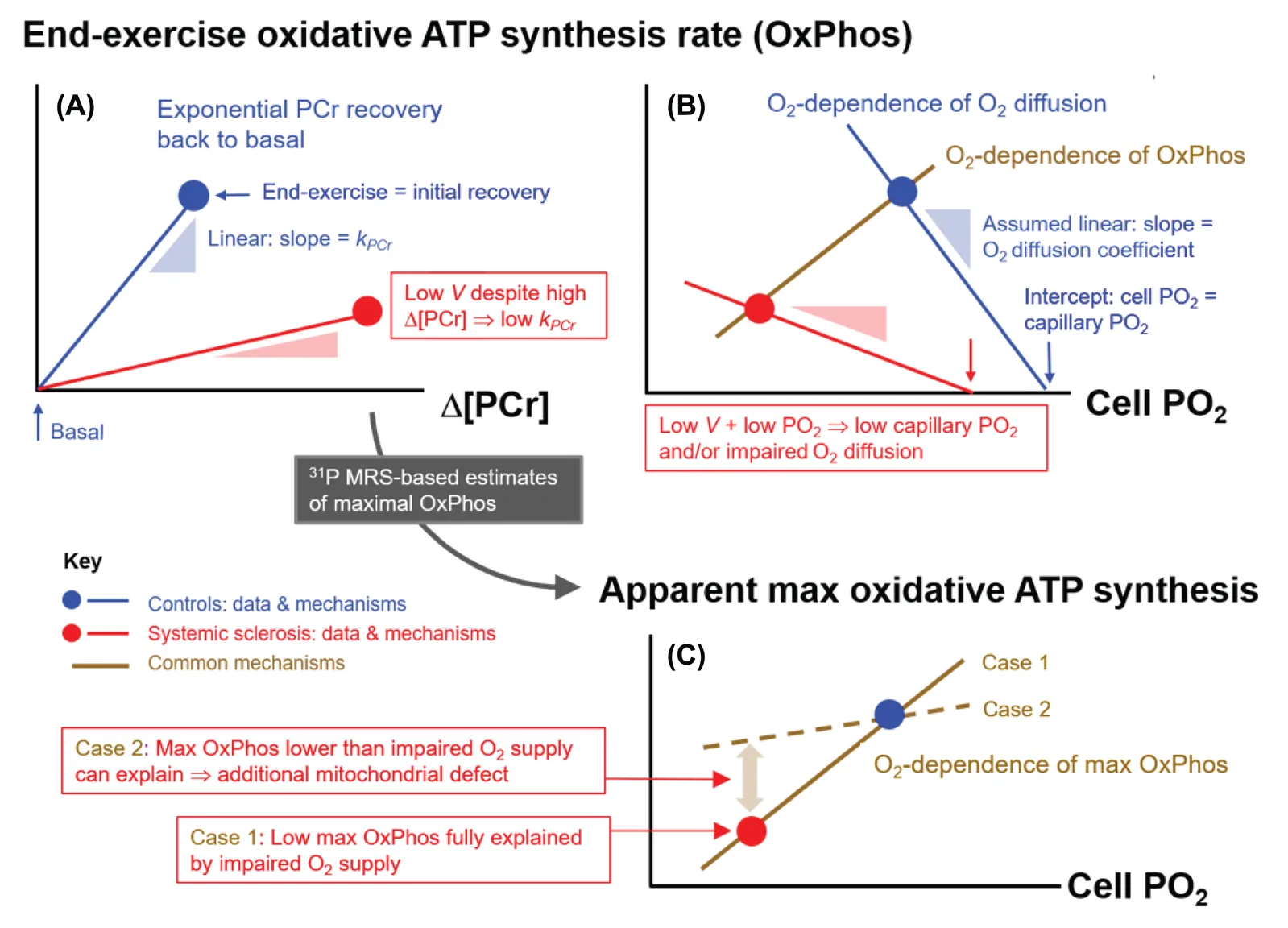
Interacting force-flux relationships in ATP and O_2_ metabolism This is a schematic illustration of physiological principles, only roughly to scale. In (**A**,**B**) the y-axis shows the rate of oxidative ATP synthesis (‘OxPhos’), the end-exercise value of which is closely approximated by the initial post-exercise rate of PCr resynthesis (*V*). This is related to the rate of O_2_ consumption by the P:O ratio, which is unlikely to be sufficiently abnormal in SSc to complicate the argument (P:O is measurable using combined ^31^P and ^1^H MRS in resting ischaemia [16], but this is challenging in patients). In (A), *V* is plotted against Δ[PCr], the fall in [PCr] below basal (resting) values; the data point is end-exercise/initial-recovery data, and monoexponential PCr recovery kinetics imply, throughout recovery, a linear relationship to the origin (i.e. the basal state) whose slope is by definition the exponential rate constant, *k*. Patient and control data are colour-coded as in the key. In (B), end-exercise *V* is plotted against end-exercise PO_2_ (if followed all the way back to basal, this would certainly be non-linear, given the different recovery kinetics of PCr and PO_2_ [8]). The negatively sloped lines represent O_2_ diffusion into the myocyte: this can be taken to be linear with muscle PO_2_ and reaches theoretical zero if muscle PO_2_ equals capillary PO_2_ [9]. The positively sloped line represents the apparent O_2_-dependence of V, assuming provisionally that PO_2_ is the only reason for the difference in *V* between the groups: this is therefore colour-coded as a common mechanism (see key). In general, at steady state (a reasonable approximation at the end of exercise), the data point should sit at the intersection of these lines (i.e. the O_2_-dependence of O_2_ transport and the O_2_-dependence of O_2_ use), such that PO_2_ is low enough to permit O_2_ to enter, but high enough to drive oxidative phosphorylation at the required rate. In (**C**) the data in Figure 2A have been used to estimate maximum values of ATP synthesis [5], plotted here against end-exercise PO_2_. The lines (Case 1 and Case 2) represent two hypothetical versions of this relationship in healthy muscle [11,12], as explained in the text. For a fuller analysis of similar issues in peripheral vascular disease, see [17].

While the initial-exercise data show no abnormality in ATP demand in SSc, the subsequent response to this involves larger changes in PCr concentration from basal (Δ[PCr]): thus, more ATP is supplied directly by PCr (and, we can infer, by anaerobic glycolysis, given the slightly larger pH fall, despite a larger PCr contribution to H^+^-buffering [3]). This is suggestive of a lower contribution by oxidative ATP synthesis, which can be directly assessed at the end of exercise, when the abrupt reduction in ATP demand redirects oxidative ATP synthesis to the restoration of PCr: this is indeed low in SSc.

^31^P MRS post-exercise recovery data are commonly used to study mitochondrial function *in vivo*, and a number of different approaches have been advocated to make inferences about the *capacity* for oxidative ATP synthesis, i.e. the notional maximum rate at maximal values of the relevant feedback signals [3]. These approaches presuppose different models of the feedback regulation principle, but are rather similar in practical terms [5]. The simplest ^31^P MRS measure in exercise where pH does not change much is simply the exponential rate constant (*k_PCr_*) of PCr recovery [5]; this can be thought of as using Δ[PCr] as a surrogate for various potential causally effective feedback signals such as Pi, ADP, and ΔG_ATP_ (which are anyway closely correlated in the absence of large pH changes). This has proved a robust measure, useful, for example, as a trial endpoint [6]. Layec and colleagues prefer to avoid committing to a particular model of mitochondrial control [4]. However, the argument can be made more abstractly, using Δ[PCr] agnostically as a feedback signal surrogate. As Figure 2A illustrates, a lower *V* despite a larger Δ[PCr] means that even increased feedback signalling is not enough to drive sufficient oxidative ATP synthesis (the conceptual point stands even though in this relatively small sample the differences in [ADP], [Pi] and ΔG_ATP_ do not reach statistical significance). In any mitochondrial model, these data imply a defect of oxidative ATP synthesis somewhere along the chain from vascular O_2_ supply to the mitochondrial O_2_ use [7]. It is a substantial defect: *V* is reduced by at least 50%, but because of the partially compensatory increase in feedback signalling, this understates the effect; a naive comparison of *Vi/Δ[PCr]* (equivalent to *k*) suggests a defect of nearer 70%, at least as large as seen in overt peripheral vascular disease [8].

As well as relating oxidative ATP synthesis to the feedback signals which match it to ATP demand, Figure 2A can be thought of as a flux-force analysis (in fact a version with ΔG_ATP_ on the x-axis is a popular way to bring non-equilibrium thermodynamics to bear on the analysis of ^31^P MRS kinetics [5]). Figure 2B takes a similar approach with the other main causal influence on oxidative ATP synthesis rate, plotting it against the ^1^H MRS-derived PO_2_. Both are lower than control in SSc: the low PO_2_ is caused by impaired O_2_ supply, and in turn limits O_2_ usage. The dashed lines in Figure 2B represent O_2_ diffusion into the myocyte [9]: the line for SSc is shown hypothetically as having both a lower slope (implying a diffusion barrier) and a lower x-intercept (implying a lower capillary PO_2_, due to impaired vascular O_2_ supply), distinguishing which will clearly need further experimental work.

It is useful to consider two hypothetical alternative results in Figure 2B. First, in a pure mitochondrial defect with no restriction in O_2_ supply one would not expect PO_2_ to be lower; indeed (by analogy with NIRS findings in mitochondrial disease [10]) PO_2_ might be higher than in controls, precisely because of the lower rate of O_2_ use.

Second, if PO_2_ were low and *V* was normal even though Δ[PCr] was high, one could conclude that low PO_2_ allowed enough O_2_ flux to sustain normal ATP synthesis, but only by a mitochondrion running with greater feedback signals. This nuance would not be visible in Figure 2B, without the feedback information implicit in Figure 2A. To capture this, we cannot avoid saying something about ‘maximal’ oxidative ATP synthesis rate. Without getting into unnecessary detail, let us posit that the analysis in Figure 2A is used somehow [3,5] to obtain maximum values, which are plotted in Figure 2C against the end-exercise PO_2_ at which they were measured. This, in very schematic form, resembles results in healthy muscle of experimental manipulation of inspired O_2_, using ^1^H MRS to measure PO_2_ and, on the y-axis, either ^31^P MRS measures of PCr recovery [11] or invasive measurements of maximal O_2_ consumption [12]. Such experiments characterise healthy mitochondria responding *in vivo* to altered muscle PO_2_: if it could be shown that the SSc results lie on the same curve as in healthy muscle (Case 1 in Figure 2C), we could be sure that impaired O_2_ supply was indeed the dominant effect; alternatively, if the SSc data lay below that line (Case 2 in Figure 2C), we would have to infer an additional component of intrinsic mitochondrial impairment. Clearly we are not yet in a position to make this distinction. We lack rich datasets on these relationships in health and disease. In another demonstration of the power of combined ^31^P/^1^H MRS methodology, this group has used it to study the O_2_-dependence of muscle mitochondrial ATP synthesis in resting ischaemia (this represents the small basal ATP turnover generally ignored in analyses of PCr recovery [5]), finding a P_50_ of 0.5 mmHg, substantially lower than some other published estimates [13]. By contrast, for maximal exercise the inspired O_2_ manipulation experiments [9,11] seem to imply a much higher P_50_, something like Case 2 in Figure 2C, and the related line of actual (as opposed to maximal) oxidative ATP synthesis rate in Figure 2B. Clearly more experimental work using combined methodologies will be necessary to resolve these issues. In the end, a comprehensive computational simulation approach [14] will likely be needed to do full justice to the complex interaction of O_2_, substrate supply and mitochondrial function in the control of oxidative ATP synthesis.

Finally, given the dominant O_2_ supply impairment in SSc, we would predict that post-exercise reoxygenation kinetics would be slower, as NIRS reveals in peripheral vascular disease [8]. This is another topic for future work.

In summary, Layec and colleagues combine ^31^P and ^1^H MRS to demonstrate a substantial impairment of oxidative synthesis in exercising skeletal muscle in patients with systemic sclerosis, and show that it is largely attributable to a defect of O_2_ supply to the mitochondria [4]. The causes of this defect now remain to be investigated.

## Open Access

Open access for this article was enabled by the participation of the University of Liverpool in an all-inclusive Read & Publish agreement with Portland Press and the Biochemical Society under an agreement with JISC.

## Abbreviations

ΔG_ATP_: free energy of ATP hydrolysis
^1^H MRS: proton magnetic resonance spectroscopy
^31^P MRS: phosphorus magnetic resonance spectroscopy
CK: creatine kinase
Cr: creatine
Mb: myoglobin
NIRS: near infrared spectroscopy
PCr: phosphocreatine
Pi: inorganic phosphate
SSc: systemic sclerosis

## References

[1] Meyerspeer M., Boesch C., Cameron D., Dezortova M., Forbes S.C., Heerschap A. et al. (2020) ^31^P magnetic resonance spectroscopy in skeletal muscle: experts’ consensus recommendations. NMR Biomed. 34, e4246 10.1002/nbm.4246 32037688 PMC8243949

[2] Fiedler G.B., Schmid A.I., Goluch S., Schewzow K., Laistler E., Niess F. et al. (2016) Skeletal muscle ATP synthesis and cellular H^+^ handling measured by localized ^31^P-MRS during exercise and recovery. Sci. Rep. 6, 32037 10.1038/srep32037 27562396 PMC4999956

[3] Kemp G.J. (2015) Muscle Studies by ^31^P MRS. eMagRes 4, 525–534

[4] Layec G., Frech T.M., Erol M.E., Machin D.R., Wray D.W., Jeong E.K. et al. (2026) Compromised muscle energetics and intracellular O_2_ availability during dynamic contraction in the plantar flexor muscles of patients with systemic sclerosis. Clin. Sci. (Lond.) 140, 1163–1174 10.1042/CS20250355 42132483 PMC13495682

[5] Kemp G.J., Ahmad R.E., Nicolay K. and Prompers J.J. (2015) Quantification of skeletal muscle mitochondrial function by ^31^P magnetic resonance spectroscopy techniques: a quantitative review. Acta Physiol. (Oxf.) 213, 107–144 10.1111/apha.12307 24773619

[6] Charles-Edwards G., Amaral N., Sleigh A., Ayis S., Catibog N., McDonagh T. et al. (2019) Effect of iron isomaltoside on skeletal muscle energetics in patients with chronic heart failure and iron deficiency. Circulation 139, 2386–2398 10.1161/CIRCULATIONAHA.118.038516 30776909

[7] Richardson R.S. (2003) Oxygen transport and utilization: an integration of the muscle systems. Adv. Physiol. Educ. 27, 183–191 10.1152/advan.00038.2003 14627616

[8] Kemp G.J., Roberts N., Bimson W.E., Bakran A., Harris P.L., Gilling-Smith G.L. et al. (2001) Mitochondrial function and oxygen supply in normal and in chronically ischemic muscle: a combined ^31^P magnetic resonance spectroscopy and near infrared spectroscopy study *in vivo*. J. Vasc. Surg. 34, 1103–1110 10.1067/mva.2001.117152 11743568

[9] Richardson R.S., Leigh J.S., Wagner P.D. and Noyszewski E.A. (1999) Cellular PO_2_ as a determinant of maximal mitochondrial O_2_ consumption in trained human skeletal muscle. J. Appl. Physiol. (1985) 87, 325–331 10.1152/jappl.1999.87.1.325 10409591

[10] Grassi B., Marzorati M., Lanfranconi F., Ferri A., Longaretti M., Stucchi A. et al. (2007) Impaired oxygen extraction in metabolic myopathies: detection and quantification by near-infrared spectroscopy. Muscle Nerve 35, 510–520 10.1002/mus.20708 17143893

[11] Haseler L.J., Hogan M.C. and Richardson R.S. (1999) Skeletal muscle phosphocreatine recovery in exercise-trained humans is dependent on O_2_ availability. J. Appl. Physiol. (1985) 86, 2013–2018 10.1152/jappl.1999.86.6.2013 10368368

[12] Richardson R.S., Grassi B., Gavin T.P., Haseler L.J., Tagore K., Roca J. et al. (1999) Evidence of O_2_ supply-dependent VO_2_max in the exercise-trained human quadriceps. J. Appl. Physiol. (1985) 86, 1048–1053 10.1152/jappl.1999.86.3.1048 10066722

[13] Erol M.E., Matias A.A., Serviente C.F., Decker S.T., Nagarajan R., Ramadan S. et al. (2026) *In vivo* quantification of mitochondrial O_2_ affinity in human skeletal muscle using ^1^H-magnetic resonance spectroscopy of deoxy-myoglobin. J. Physiol. 604, 4876–4888 10.1113/JP290548 42178176 PMC13267685

[14] Beard D.A. (2005) A biophysical model of the mitochondrial respiratory system and oxidative phosphorylation. PLoS Comput. Biol. 1, e36 10.1371/journal.pcbi.0010036 16163394 PMC1201326

[15] Boesch C. and Kreis R. (2016) Muscle Studies by ^1^H MRS. eMagRes 5, 1097–1108

[16] Erol M.E., Bannon S.T., Matias A.A., Siokas T., Nagarajan R., Fur Y.L. et al. (2025) Mitochondrial efficiency in resting skeletal muscle *in vivo:* a novel non-invasive approach using multinuclear magnetic resonance spectroscopy in humans. J. Physiol. 603, 1503–1519 10.1113/JP287412 39960635 PMC11908483

[17] Kemp G.J. (2004) Mitochondrial dysfunction in chronic ischemia and peripheral vascular disease. Mitochondrion 4, 629–640 10.1016/j.mito.2004.07.017 16120420

